# Chromosome painting in plants: history and future perspectives

**DOI:** 10.1007/s10577-026-09818-1

**Published:** 2026-09-18

**Authors:** Jiming Jiang

**Affiliations:** Department of Plant Biology, Department of Horticulture, Michigan State University AgBioResearch, Michigan State University, East Lansing, MI 48824 USA

**Keywords:** Oligo-FISH, Chromosome rearrangement, Karyotype, Chromosome evolution

## Abstract

Chromosome painting was developed in mammalian species nearly four decades ago and rapidly became a powerful tool for chromosome identification, comparative cytogenetics, and evolutionary genome analysis. Comparative chromosome painting among diverse mammals generated much of the foundational knowledge of chromosome structure, chromosomal rearrangements, and karyotype evolution before the advent of whole-genome sequencing. Although chromosome painting was first demonstrated in plants in 2001, its applications remained largely restricted to a few plant lineages until the development of oligonucleotide (oligo)-based chromosome painting in 2015. During the last decade, oligo-based chromosome painting has transformed plant cytogenetics, enabling many investigations that were previously impossible. These studies have provided new insights into meiotic chromosome pairing, crossover formation, chromosome fusion, karyotype stability, and chromosome evolution across diverse plant lineages. This review summarizes the history of technological development of chromosome painting in plants, highlights major discoveries enabled by oligo-based chromosome painting, and discusses future opportunities, particularly the integration of chromosome painting with three-dimensional chromosome and genome biology.

## Development of chromosome painting in mammalian species

The chromosome painting (CP) methodology was developed nearly 40 years ago and represented a major breakthrough in molecular cytogenetics (Lichter et al. 1988; Pinkel et al. 1988). In these pioneering studies, a large number of lambda phage or plasmid clones specific to a single human chromosome were pooled and labeled as a probe for fluorescence in situ hybridization (FISH). The resulting probes produced uniform hybridization signals along the entire length of the target chromosome, enabling the direct visualization and identification of individual chromosomes in metaphase spreads or interphase nuclei (Lichter et al. 1988; Pinkel et al. 1988). Some phage or plasmid clones contained dispersed repetitive DNA elements, which generated cross-hybridization signals on non-target chromosomes. This problem was overcome by suppression hybridization, in which an excess of sheared and unlabeled human genomic DNA was included during probe preparation to block repetitive sequences shared among chromosomes, thereby greatly enhancing the signal quality and chromosome specificity (Lichter et al. 1988; Pinkel et al. 1988).

Soon thereafter, chromosome-specific DNA was generated directly from individual chromosomes isolated by flow cytometry or chromosome microdissection. The minute amounts of chromosomal DNA were amplified using degenerate oligonucleotide-primed PCR (DOP-PCR) (Meltzer et al. 1992; Telenius et al. 1992) or related amplification methods. The amplified DNA was then labeled for FISH. These technological advances greatly expanded the application of CP to a broad range of mammalian species (Yang et al. 1999) and later to non-mammalian vertebrates, including birds, reptiles, and fishes (Giovannotti et al. 2009; Griffin et al. 1999; Nanda et al. 2006; Shetty et al. 1999; Zimmer et al. 1997) and insects (Fuchs et al. 1998).

Clinical cytogenetics was among the earliest and most important beneficiaries of the CP methodology because it provided an effective means of identifying aneuploidies and rearranged human chromosomes (Ried et al. 1998). CP also became a powerful research tool, especially in evolutionary cytogenetics through comparative chromosomal painting (CCP), also known as cross-species chromosome painting or Zoo-FISH (Jauch et al. 1992; Wienberg et al. 1990). By directly visualizing conserved chromosomes or chromosomal segments among related species, CCP enabled the identification of shared syntenic blocks, chromosomal rearrangements, and evolutionary breakpoints, providing unprecedented insights into karyotype evolution across diverse vertebrate lineages (Ferguson-Smith and Trifonov 2007; Graphodatsky et al. 2011; Wienberg and Stanyon 1997). Extensive CCP analyses across a wide range of mammalian taxa, including marsupial and eutherian species, provided much of the foundational knowledge on chromosomal relationships and karyotype evolution among mammalian species prior to the genome sequencing era (Ferguson-Smith and Trifonov 2007; Graphodatsky et al. 2011).

## Early attempts to develop chromosome painting in plants

The striking CP images obtained in mammalian species and the tremendous potential of CP as a new cytogenetic tool stimulated several plant laboratories to adapt the methodology for plant research. These efforts were further encouraged by the successful establishment of both chromosome sorting and chromosome microdissection in plants (Dolezel et al. 1994; Houben et al. 1996). However, probes developed from sorted or microdissected plant chromosomes encountered a major obstacle: they contained a high proportion of repetitive DNA sequences that generated extensive cross-hybridization signals throughout the genome. The suppression hybridization protocol using unlabeled genomic DNA from the target species was insufficient to achieve chromosome-specific labeling. This challenge was clearly demonstrated by Ingo Schubert and his colleagues (Fuchs et al. 1996). They generated painting probes from microdissected chromosomes of three species, *Vicia faba*, *Picea abies*, and *Triticum aestivum*, all of which possess large genomes enriched in repetitive DNA. Extensive cross-hybridization was observed in all three species, preventing the observation of chromosome-specific signals (Fuchs et al. 1996).

I joined the University of Wisconsin-Madison in June 1995 as a plant cytogeneticist and immediately initiated a collaboration with Dr. K. (Aru) Arumuganathan. Aru was one of the pioneers of plant flow cytometry (Arumuganathan and Earle 1991) and managed the Flow Cytometry Core Facility at the University of Nebraska-Lincoln. Using flow cytometry, Aru successfully sorted the largest chromosome from both rice (*Oryza sativa*) and tomato (*Solanum lycopersicum*). We developed FISH probes from the sorted chromosomal DNA; however, the probes produced hybridization signals on all rice and tomato chromosomes rather than on a single target chromosome. This result was particularly disappointing considering the fact that rice and tomato possess substantially smaller genomes than the species analyzed by Fuchs et al. (Fuchs et al. 1996). We made numerous attempts to optimize both DOP-PCR and suppression hybridization conditions, but none of these efforts overcame the extensive cross-hybridization problem.

Prior to my CP experiments, I had gained FISH experience using bacterial artificial chromosome (BAC) clones in sorghum (*Sorghum bicolor*) (Woo et al. 1994) and rice (Jiang et al. 1995). A substantial proportion of sorghum and rice BACs generated weak to very strong hybridization signals across all chromosomes. Although the application of unlabeled sorghum and rice genomic DNA, commonly referred to as “blocking DNA”, could reduce the background associated with BACs producing relatively weak cross-hybridization signals, it was largely ineffective for BACs generating strong genome-wide hybridization. Based on these observations, together with the unsuccessful attempts using sorted chromosomes in rice and tomato, I concluded that it would be extremely difficult, if not impossible, to develop CP in plants based on traditional probes derived from flow-sorted or microdissected chromosomes, due to the overwhelming abundance of repetitive DNA in most plant genomes. Surprisingly, chromosome painting using microdissection-derived probes has recently been reported in maize and pepper (Soares et al. 2020), although the methodology has become outdated due to the emergence of oligonucleotide (oligo)-based painting probes (see below).

## BAC-based chromosome painting, the first practical solution in plants

After a decade of unsuccessful attempts to adapt CP to plants, the plant cytogenetics community welcomed the first major breakthrough in 2001. Martin Lysak, Ingo Schubert, and colleagues demonstrated that chromosome painting could be achieved by pooling a large number of repeat-free BACs into a single FISH probe in the model plant *Arabidopsis thaliana* (2n = 10) (Lysak et al. 2001). This methodology benefited from two key genomic features of *A. thaliana*. First, *A. thaliana* possesses a very small genome of ~ 125 Mb (The Arabidopsis Genome Initiative 2000). Consequently, a relatively small number of BACs, each containing inserts of approximately 100–200 kb, can collectively cover an entire chromosome. Second, the *A. thaliana* genome is highly euchromatic, with most repetitive DNA concentrated in the centromeric and pericentromeric regions. As a result, the majority of BAC clones contain little or no repetitive DNA and therefore do not generate significant cross-hybridization signals.

As a reviewer of the original Lysak et al. manuscript, I was among the first scientists to read this exciting study. I immediately recognized both the potential and the limitations of this strategy. First, the painting probes developed in *A. thaliana* would provide a powerful CCP tool for studying chromosome evolution across the Brassicaceae. This expectation was based in part on our own experience using *A. thaliana* BACs for FISH mapping in *Brassica* species (Jackson et al. 2000)*.* Second, the success of this strategy depended on the availability of repeat-free BACs distributed throughout the chromosome. Based on our previous FISH mapping of BACs in sorghum (Woo et al. 1994)*,* rice (Cheng et al. 2001; Jiang et al. 1995)*,* and potato* (Solanum tuberosum)* (Dong et al. 2000; Song et al. 2000)*,* all of which possess relatively small genomes, a substantial proportion of BAC clones from these species nevertheless contained repetitive sequences and generated extensive cross-hybridization signals. Consequently, a lower proportion of BACs from these species would be suitable for CP applications. I therefore anticipated that the BAC-based strategy would only be applicable to plant lineages containing model species with exceptionally small and highly euchromatic genomes similar to that of *A. thaliana*. Indeed, the methodology was later adopted in *Brachypodium distachyon* (Idziak et al. 2011), another species with a relatively small genome (approximately 272 Mb) and a high proportion of euchromatic DNA (The International Brachypodium Initiative 2010).

Since the development of BAC-based chromosome painting, Martin Lysak and colleagues have conducted extensive comparative genome analyses across numerous Brassicaceae species using *A. thaliana* BAC-derived painting probes. These studies have generated fundamental insights into genome dynamics and karyotype evolution within the family, including chromosome triplication, descending dysploidy, chromosome fusion and fission events, and centromere repositioning (Lysak et al. 2006, 2005; Schubert and Lysak 2011). Collectively, this body of work established CP as a powerful approach for investigating plant genome evolution and provided a conceptual framework for CP-based chromosome studies in other plant lineages.

## Fluorescent-labeled oligo pools, an untold chromosome painting story in plants

**Fig. 1:**
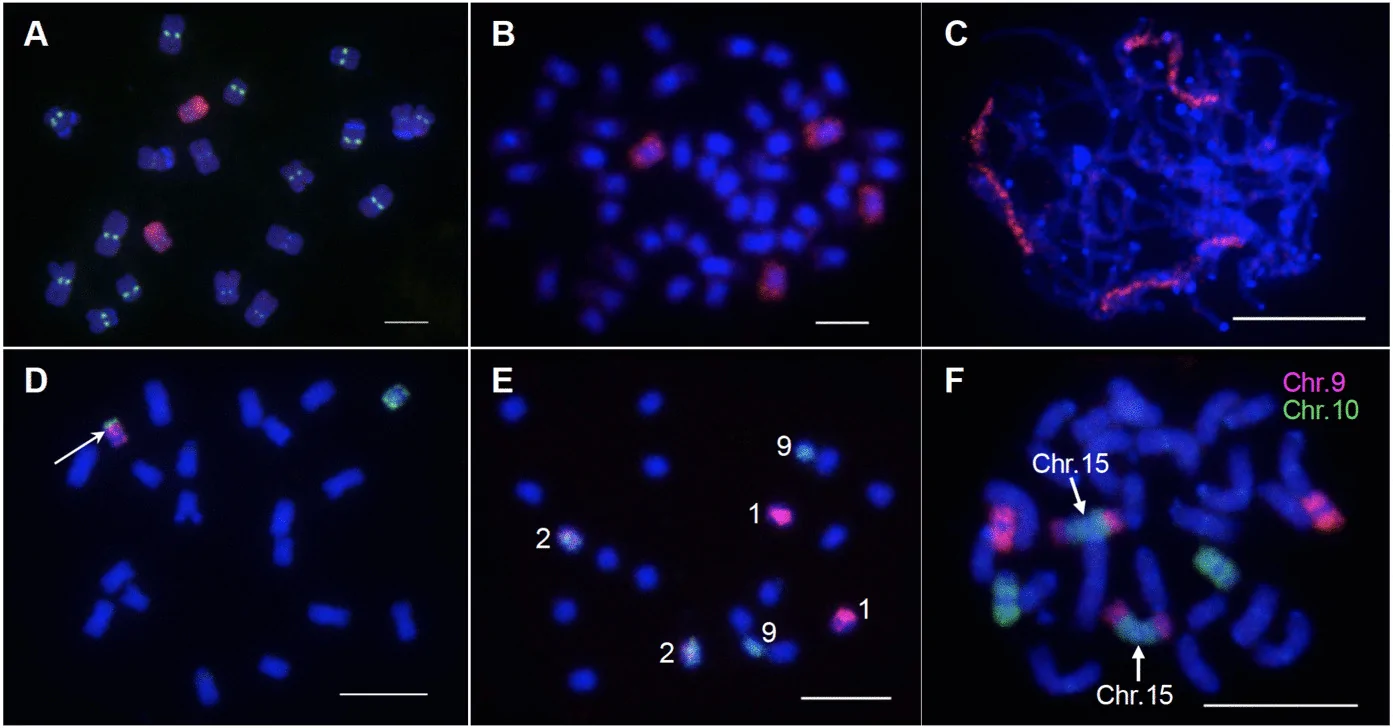
Representative applications of oligo-based chromosome painting in plants. **A** Chromosome painting in maize using a fluorescent-labeled oligo pool designed from exonic sequences of maize chromosome 8. Centromeres of all maize chromosomes are detected with the centromeric satellite repeat CentC. **B** Chromosome painting in the wild tetraploid rice *Oryza minuta* (2n = 4x = 48, BBCC) using a fluorescent-labeled oligo pool designed from exonic sequences of rice chromosome 8. Painting signals are detected on the two homoeologous chromosomes 8 (8B and 8C). **C** Chromosome pairing of the six copies of chromosome 11 in *Solanum demissum* (2n = 6x = 72) at the pachytene stage of meiosis. The six homologs form three bivalents. **D** Detection of a meiotic crossover on maize chromosome 10 using haplotype-specific chromosome painting. Chromosomes 10 from Mo17 and B73 are painted green and red, respectively. The arrow indicates a crossover on a recombinant chromosome 10. (**E**) Comparative chromosome painting in *Boenninghausenia albiflora* (2n = 20) using three chromosome-painting probes for chromosomes 1 (red), 9 (green), and 2 (red-green mix) developed from *Citrus maxima* (2n = 18)*.* **F** Chromosome 15 (arrows) in *Erianthus rockii* (2n = 4x = 30) is derived from the fusion of ancestral chromosomes 9 (red) and 10 (green). Scale bars = 5 μm (A, B, E) and 10 μm (C, D, F)

Massively parallel synthesis of large numbers of oligos became technically possible in the early 2000s, when thousands of independent DNA oligos could be synthesized simultaneously on microarrays and subsequently recovered as complex sequence pools (Tian et al. 2004). These oligo pools could be fluorescently labeled, creating a new class of highly customizable hybridization probes. My long-time collaborator Jim Birchler was one of the first scientists to envision the application of fluorescent-labeled oligo pools as chromosome painting probes. As early as 2010, Jim collaborated with NimbleGen (later acquired by Roche), a biotechnology company based in Madison, Wisconsin, to develop several chromosome-specific oligo pools. For each pool, oligo sequences were designed from the exons of genes located on a single maize chromosome. Remarkably, these oligo pools generated strong and highly specific painting signals along the target maize chromosomes (Fig. 1A).

Jim shared these chromosome-painting images during one of our project meetings. I was struck by the quality of the images and by the possibility that fluorescent-labeled oligo pools could become a broadly applicable chromosome-painting strategy for plants. Motivated by Jim’s results, I initiated a collaboration with NimbleGen colleagues to test the approach in rice. We identified all annotated exons on rice chromosome 8, a chromosome we had worked on for several years (Nagaki et al. 2004; Yan et al. 2005), and retained only those lacking detectable paralogs elsewhere in the rice genome. The resulting chromosome 8-specific exon pool generated highly specific hybridization signals with virtually no detectable cross-hybridization to other rice chromosomes. The probe painted a single chromosome pair in all diploid *Oryza* species and two pairs in wild tetraploid rice species (Fig. 1B), demonstrating its utility for comparative cytogenetic studies.

Despite its technical success, the methodology faced a significant practical limitation: cost. At the time, synthesis of approximately 10 µg of chromosome 8-specific exon pool cost US$3,000. Approximately 200 ng was required per slide to generate sufficiently strong hybridization signals, resulting in a cost of roughly US$60 per experiment. Such costs were difficult to justify for routine cytogenetic applications and greatly limited the scalability of the approach. Consequently, neither Jim nor I ultimately published these studies. As a result, this early exploration of fluorescent-labeled oligo pools as chromosome-painting probes remains an untold chapter in the history of plant chromosome painting. Nevertheless, this methodology was eventually reported in a mammalian paper, co-authored by NimbleGen scientists (Boyle et al. 2011).

## PCR-based oligo pools, a true revolution in chromosome painting

In 2012, a relatively simple modification of oligo-pool design proved to be a turning point in the development of chromosome painting, particularly for non-model species with limited genomic resources (Beliveau et al. 2012). In this system, every oligo within a pool is flanked by common primer sequences that enable PCR-based amplification and subsequent fluorescent labeling (Beliveau et al. 2012). As a result, the minute quantity of oligos obtained from commercial synthesis can serve as a renewable source of FISH probes. This innovation effectively eliminated the cost barrier associated with directly fluorescent-labeled oligo pools and made large-scale chromosome painting applications economically feasible.

We became the first group to adapt this strategy for chromosome painting in plants using cucumber (*Cucumis sativus*) as a model (Han et al. 2015). Our study demonstrated several key features of this new chromosome painting technique. First, this methodology, in principle, can be applied to any plant species with a sequenced genome. Thus, for the first time, the plant research community had access to a broadly applicable chromosome-painting technology. Second, the painting probes developed from cucumber successfully painted partially homologous chromosomes in related *Cucumis* species that diverged from cucumber up to approximately 12 million years ago, demonstrating the power of the painting probes for CCP studies. Third, because the oligo pools can be amplified nearly indefinitely using the primers associated with each oligo, a single synthesis reaction can support an enormous number of FISH experiments. Consequently, once developed, each oligo-based probe becomes a long-term community resource that can be shared among laboratories. Finally, we developed Chorus, a software package for oligo identification and probe design in plants. Chorus was later upgraded to Chorus2 (Zhang et al. 2021), which has become a widely used tool for oligo-FISH probe design in plants.

## Major applications of oligo-based chromosome painting in plants

The introduction of PCR-amplifiable oligo pools fundamentally transformed chromosome painting in plants. Since our first report in 2015 (Han et al. 2015), oligo-based chromosome painting has been rapidly adopted across a broad spectrum of plant species, demonstrating its versatility in both basic and applied research. The methodology has been successfully applied to major food crops, including rice (Hou et al. 2018), maize (Albert et al. 2019; Martins et al. 2019), barley and wheat (Li et al. 2021; Shi et al. 2022; Song et al. 2020), potato (Braz et al. 2018; He et al. 2018), sweetpotato (Sun et al. 2025, 2022), and dry beans (Dias et al. 2024; Martins et al. 2021); horticultural crops, including strawberry (Qu et al. 2021, 2017), cucumber (Zhao et al. 2021, 2019), banana (Beránková et al. 2024; Simoníková et al. 2020; Simonikova et al. 2019), and citrus (He et al. 2026b, 2020); industrial crops, including poplar (Xin et al. 2018, 2020), cotton (Liu et al. 2020b), peanut (Li et al. 2024), oil palm (Zaki et al. 2021), and sugarcane (Meng et al. 2023, 2021; Piperidis and D'Hont 2020); as well as numerous model and non-model plant species used for diverse biological investigations (Agrawal et al. 2020; Bacovsky et al. 2020; Beying et al. 2020; Bielski et al. 2020; Chen et al. 2023, 2024; Filiault et al. 2018; He et al. 2026a; Hoang et al. 2021; Kuo et al. 2025; Meng et al. 2024; Wang et al. 2026).

Remarkably, many of these species were traditionally considered unsuitable for cytogenetic studies because of their extremely small chromosomes, a large number of chromosomes, or complex polyploid genomes. Consequently, cytogenetic analyses in these species were often limited or rarely attempted. Oligo-based chromosome painting has extended modern molecular cytogenetics to these challenging systems because it enables the effective identification and analysis of specific chromosomes regardless of their complex cytogenetic backgrounds. As a result, the new chromosome-painting methodology has created unprecedented opportunities to investigate the structure, transmission, and evolution of individual chromosomes, as well as the organization and evolution of entire genomes, in a wide range of plant species. Below, I summarize the major applications and achievements of oligo-based plant chromosome painting since 2015.

### Chromosome identification and karyotyping

Reliable identification of individual chromosomes is the foundation of cytogenetic research. Only a few plant species, including maize and wheat, became classical models for cytogenetic studies, largely because chromosome identification systems were established early in these species. In maize, chromosome identification relied on the distinct morphology of its meiotic pachytene chromosomes (Creighton and McClintock 1931; McClintock 1930), whereas in wheat it was facilitated by the availability of multiple sets of aneuploid stocks associated with individual chromosomes (Sears 1954, 1969).

Chromosome banding and FISH represented two major modern approaches for chromosome identification. However, only a limited number of plant species benefited from chromosome banding techniques (Friebe et al. 1996a; Guerra et al. 2000). Although FISH proved to be more broadly applicable in plants, FISH-based chromosome identification depends on the availability of robust probes specific to individual chromosomes. Developing such probes turned out to be a major challenge, particularly for non-model species lacking extensive genomic resources (Jiang and Gill 2006). Consequently, reliable chromosome identification systems based on FISH were established in only a small number of plant species (Jiang and Gill 2006).

The most direct and universal application of chromosome painting is the highly reliable identification of individual chromosomes. Once developed, a chromosome-painting probe allows the target chromosome to be unambiguously identified and tracked throughout different stages of mitosis and meiosis (He et al. 2018). More importantly, CP-based chromosome identification is largely independent of chromosome size and not compromised by complex cytogenetic background. This advantage is best exemplified by studies in sugarcane. Modern sugarcane cultivars (2n = 100–120) are highly complex polyploids and aneuploids derived from interspecific hybrids between *Saccharum officinarum* (2n = 8x = 80) and *Saccharum spontaneum* (2n = 40–128). Despite this extraordinary cytogenetic complexity, all sugarcane chromosomes can be traced to ten ancestral chromosomes (Piperidis and D'Hont 2020). Consequently, a chromosome-painting probe designed from one ancestral chromosome enables effective tracking of both the intact chromosome and its rearranged derivatives in modern sugarcane cultivars and related *Saccharum* species (Meng et al. 2021; Piperidis and D'Hont 2020; Wang et al. 2022a; Yu et al. 2022, 2025). In addition to probes painting an entire chromosome, barcode painting probes, which generate regional signals on multiple chromosomes, enable the identification of all chromosomes in a single FISH experiment (Jiang 2019). Barcode painting probes have been developed in diverse plant species for chromosome identification (Agrawal et al. 2020; Braz et al. 2018, 2020; Bustamante et al. 2021; Chen et al. 2020; Dolezalová et al. 2022; Harun et al. 2024; Liu et al. 2020a; Mata-Sucre et al. 2024; Meng et al. 2022, 2025; Nascimento and Pedrosa-Harand 2023; Wang et al. 2022b; Zaki et al. 2021).

Karyotypes have been reported for a large number of plant species. However, most published karyotypes were not based on individually identified chromosomes. In the traditional karyotyping procedures, chromosomes were measured and arranged solely according to their length and arm ratio. As a result, true karyotypes consisting of individually identified chromosomes were established in only a few model plant species. Because chromosome-painting probes can now be readily developed for every chromosome in a target species (Albert et al. 2019; Chen et al. 2023, 2024; He et al. 2020; Meng et al. 2024; Shi et al. 2022; Simonikova et al. 2019; Wang et al. 2026; Xin et al. 2020), chromosome painting has made it possible to establish authentic karyotypes with individually identified chromosomes in a wide range of plants. Such karyotypes provide a robust cytogenetic framework for comparative and evolutionary studies.

### Chromosome pairing during meiosis

Identification of individual chromosomes at prophase I and metaphase I (MI) of meiosis is technically much more demanding than chromosome identification at mitotic metaphase. Consequently, reliable meiotic chromosome identification has been achieved in only a few model plant species. For example, individual wheat and rye chromosomes at MI can be identified using chromosome banding techniques (Curtis et al. 1991; Naranjo et al. 1987). One major advantage of CP-based chromosome identification is that a target chromosome can be unambiguously tracked through nearly all stages of meiosis (He et al. 2018). This capability has enabled detailed analyses of chromosome behavior during cell division, particularly chromosome pairing during meiosis.

The extent of chromosome pairing has traditionally been used to infer homology among chromosomes from different subgenomes in polyploid species. Exclusive bivalent pairing is expected in classical allopolyploids composed of distinct subgenomes, whereas multivalent formation is expected in autopolyploids. However, it is often difficult to unambiguously identify the various multivalent configurations in most polyploid plants. In addition, traditional pairing analyses provide only the average frequencies of chromosome configurations in pollen mother cells (PMCs) and do not reveal the behaviors of individual chromosomes. Consequently, the classification of many polyploids as allopolyploids, autopolyploids, or segmental allopolyploids has remained controversial.

*Solanum demissum* (2n = 6x = 72), a wild hexaploid extensively used in potato breeding, provides an excellent example. Based on classical chromosome-pairing analyses, this species was proposed to contain two similar subgenomes that differ from a third subgenome (AABBBB or AABBB^1^B^1^) (Hawkes 1958; Irikura 1976; Marks 1955). We analyzed MI chromosome pairing in *S. demissum* using painting probes specific to potato chromosomes 2, 4, 7, and 11. Exclusive bivalent pairing was observed in 93% of PMCs, ranging from 83.2% for chromosome 2 to 98.4% for chromosome 11 (He et al. 2018) (Fig. 1C). Furthermore, no hexavalents were observed in meiosis. Similarly, chromosome painting revealed predominantly bivalent pairing in several autopolyploid clones of *Saccharum spontaneum* (Zhang et al. 2023).

In contrast, cultivated sweetpotato (*Ipomoea batatas*) had long been proposed to be either an autohexaploid or an allohexaploid based on classical chromosome-pairing analyses (Magoon et al. 1970; Nishiyama 1971; Ting et al. 1957). A recent chromosome-painting study unambiguously demonstrated the presence of high frequencies of hexavalents and quadrivalents, observed in more than 90% of PMCs, in both *I. batatas* and its wild progenitor *Ipomoea trifida* (6x), strongly supporting their autopolyploid nature (Sun et al. 2022). Thus, chromosome-painting-based analyses of additional autopolyploids and segmental autopolyploids should provide important insights into the frequency and mechanisms of meiotic adaptation leading to predominantly bivalent pairing in natural polyploids (Bomblies et al. 2016).

Wild species are widely used as sources of valuable germplasm for crop improvement (Friebe et al. 1996b; Jansky 2000). The successful introgression of beneficial alleles from wild species depends largely on recombination between homologous or homoeologous chromosomes in interspecific hybrids. Such recombination potential can be assessed through analyses of chromosome pairing during meiosis. Chromosome painting enables the direct tracking of parental chromosomes throughout meiosis in these hybrids, allowing the pairing behavior of homologous and homoeologous chromosomes to be visualized and their recombination potential to be evaluated (Han et al. 2015).

### Haplotype-specific chromosome painting

In comparative chromosome painting using probes developed from a model species, the intensity of FISH signals on chromosomes generally decreases as the target species becomes more distantly related to the source species. However, an important question remained unanswered: what level of sequence divergence between an oligo and its target sequence can still support efficient hybridization under standard FISH conditions?

To address this question, we developed a series of oligo pools based on the chromosome 10 sequences from two maize inbreds, B73 and Mo17. The sequence of each oligo (45 bp) within a pool was designed from either B73 or Mo17 and differed from the corresponding sequence in the other genotype by 1, 2, 3–4, and ≥ 5 single-nucleotide polymorphisms (SNPs) (Martins et al. 2019). FISH experiments using these painting probes led to two important discoveries. First, a B73 (or Mo17) probe consisting of oligos containing a minimum of 3–4 SNPs relative to the corresponding Mo17 (or B73) sequence generated strong signals on chromosome 10 of the source genotype but significantly weaker signals on chromosome 10 of the other genotype. Such probes therefore function as haplotype-specific painting probes, enabling differential visualization of the two homologous copies of chromosome 10 within the same cell (Martins et al. 2019). Second, a painting probe consisting of only approximately 5,000 oligos was sufficient to generate painting signals on maize chromosome 10, which spans approximately 151 Mb of DNA (Schnable et al. 2009; Sun et al. 2018). Based on these two findings, namely, the level of sequence divergence required for haplotype discrimination and the relatively small number of oligos needed for effective chromosome painting, we anticipated that haplotype-specific painting probes could be readily developed in other plant species. Indeed, haplotype-specific painting probes have recently been demonstrated for sugarcane chromosomes 8A, 8B, 8C, and 8D in an octoploid *Saccharum spontaneum* accession (Meng et al. 2023) and for four different haplotypes of chromosome 5H in tetraploid *Hordeum bulbosum* (Feng et al. 2025).

For the first time, haplotype-specific painting probes enabled the cytological differentiation of true homologous chromosomes within the same cell, opening the door to a wide range of biological questions that were previously inaccessible to cytogenetic analysis. The two maize probes allowed direct visualization of meiotic crossovers (COs) on recombinant chromosome 10 in an F_2_ population derived from a B73 x Mo17 hybrid (Fig. 1D). Analysis of a large number of recombinant chromosomes revealed the distribution of COs along the entire length of chromosome 10 and the size of the recombination-suppressed domain spanning the centromere. In addition, haplotype-specific painting in recombinant inbred lines derived from an intermated B73 x Mo17 population detected COs within the centromeric region, suggesting that recombination events can accumulate in chromosomal regions that are normally recombination-suppressed (Martins et al. 2019).

Although meiotic chromosome pairing has been extensively investigated in polyploid plants, the extent to which DNA sequence variation contributes to the establishment of exclusive homologous chromosome pairing has remained unclear. The haplotype-specific probes developed for maize chromosome 10 allowed us to address this question directly. We generated polyploid maize plants containing three copies of chromosome 10. We found that the two identical chromosome 10 homologs derived from inbred H99 pair preferentially over chromosome 10 from inbred B73 at multiple stages of meiosis. These results demonstrated that homologous chromosome pairing preferentially occurs between partners with the highest sequence similarity and that chromosome pairing can discriminate among chromosomes based on cryptic sequence variation (Braz et al. 2021). Thus, an innate preference of homologous chromosome pairing exists in nascent allopolyploids and may represent the first layer of discrimination that ultimately suppresses homoeologous chromosome pairing in established allopolyploids (Braz et al. 2021).

### Chromosome evolution revealed by comparative chromosome painting

CCP among evolutionarily related species has been one of the most important applications of chromosome painting. CCP enables the direct visualization of conserved syntenic chromosomal segments or entire chromosomes between the probe-source species and a target species, thereby revealing evolutionary relationships at the chromosome level. Lysak and colleagues pioneered CCP studies between *A. thaliana* and a large number of Brassicaceae species, generating fundamental insights into chromosome and genome evolution within the family (Lysak et al. 2006, 2005, 2003). The development of oligo-based chromosome painting has opened the door for CCP studies in numerous additional plant lineages. Below, I summarize some of the major insights that have emerged from oligo-based CCP.

#### The limits of painting distantly related species

The success of comparative chromosome painting depends on the degree of sequence homology between the probe sequences and their targets during cross-species hybridization. BAC-based painting probes developed from *A. thaliana* have been successfully used in CCP analyses across a large number of Brassicaceae species that diverged up to ~ 20 million years ago (Mya) (Hlousková et al. 2019; Mandáková et al. 2017).

Oligo-based chromosome painting probes have exhibited variable levels of success in different plant lineages. Probes developed in cucumber successfully labeled homoeologous chromosomes in related *Cucumis* species that diverged up to 12 Mya but failed to generate signals on chromosomes from more distantly related taxa (Han et al. 2015). In contrast, two painting probes developed from *Ipomoea nil* generated strong signals in 16 related species that diverged over a period of approximately 25 million years (Sun et al. 2025). More recently, we demonstrated that painting probes developed from the model citrus species *Citrus maxima* successfully labeled chromosomes of *Boenninghausenia albiflora* (Fig. 1E), a Rutaceae species that diverged from *C. maxima* more than 50 Mya (He et al. 2026a).

These examples demonstrated that the phylogenetic distance provides only a general prediction of the utility of chromosome painting in distantly related species. The true determinant of successful cross-species painting is the extent of DNA sequence divergence between the source and target genomes. Despite their deep evolutionary divergence, *B. albiflora* and *C. maxima* possess chromosomes of similar size, suggesting that their genome sizes and overall genome compositions have remained relatively stable. In contrast, probes developed from *C. maxima* generated poor signals on chromosomes from Rutaceae species with substantially larger chromosomes (Li He, unpublished). We speculate that these genomes have undergone more extensive transposable element amplification and genome expansion, resulting in accelerated sequence divergence and reduced hybridization efficiency. Therefore, species with relatively small and highly euchromatic genomes may serve as ideal source species for probe design because their painting probes are more likely to retain utility across broader phylogenetic distances.

#### Exceptionally stable karyotypes in woody plants

Extensive CCP-based investigations in the Brassicaceae revealed highly dynamic patterns of chromosome and karyotype evolution within the family (Lysak and Lexer 2006; Mandáková and Lysak 2008). In contrast, recent CCP studies have uncovered extraordinarily stable chromosome synteny and karyotypes in two independent lineages of woody plants.

*Populus* is a genus of dioecious woody plants in the Salicaceae family and consists predominantly of diploid species with a basic chromosome number of n = 19. Because *Populus* chromosomes are relatively small and morphologically similar, a karyotype with individually identified chromosomes had never been established. Xin et al. (2020) developed a complete set of 19 chromosome-painting probes, enabling the identification and tracking of every chromosome in five *Populus* species that diverged approximately 14 million years ago (Xin et al. 2020). Remarkably, no interchromosomal structural rearrangements were detected in any of the 19 chromosomes among these species. Thus, both chromosome synteny and karyotype structure have remained highly conserved during the evolution of the genus.

We recently conducted CCP studies in 13 woody species belonging to the Aurantioideae subfamily of the Rutaceae, including pomelo (*C. maxima*, 2n = 2x = 18). A complete set of nine chromosome-painting probes was developed from *C. maxima* (He et al. 2020). Similar to the observation in *Populus*, all 13 Aurantioideae species retained complete chromosomal synteny and highly similar karyotypes despite approximately 20 million years of divergence (He et al. 2026b).

Both *Populus* and Aurantioideae are composed of perennial woody species. It is well established that perennial woody plants exhibit substantially slower rates of molecular evolution than herbaceous plants, a pattern generally attributed to their long generation times (Gaut et al. 1996; Kay et al. 2006; Smith and Donoghue 2008). The molecular evolutionary rate in *Populus* was estimated to be approximately one-sixth that of *A. thaliana* (Tuskan et al. 2006). We therefore hypothesize that both the molecular clock and the chromosomal clock run more slowly in woody plants. Woody species may require considerably longer periods of time to generate, establish, and fix chromosomal rearrangements or changes in chromosome number than annual herbaceous plants.

Testing this hypothesis will require comparative chromosome-painting analyses across a broader range of both woody and herbaceous lineages. The emerging evidence of exceptionally stable karyotypes in woody plants underscores the need for additional comparative studies, which will be essential for understanding the factors that determine the rates of chromosomal and karyotypic evolution in plants.

#### Mechanisms and genomic consequences of chromosome fusions

Chromosome fusions played a major role in karyotype evolution within the Brassicaceae (Jiang et al. 2025; Lysak 2022; Mandáková and Lysak 2018). Although numerous chromosome fusion events have been identified through CCP and genome sequencing, little is known about the mechanisms responsible for these fusions or their long-term genomic consequences.

More recently, chromosome fusions have been discovered in several members of the *Saccharum* complex, including sugarcanes (Yu et al. 2022). The ancestral genome of the *Saccharum* complex is inferred to have consisted of ten basic chromosomes. In two allotetraploid species, *Narenga porphyrocoma* and *Erianthus rockii* (2n = 4x = 30), one subgenome retains all ten basic chromosomes. In contrast, the second subgenome contains only five chromosomes, each resulting from the fusion of two ancestral chromosomes (Fig. 1F). Using a high-resolution regional painting (HRP) approach, we performed detailed analyses of these fusion chromosomes and gained new insights into their origin and their genomic consequences.

**Fig. 2:**
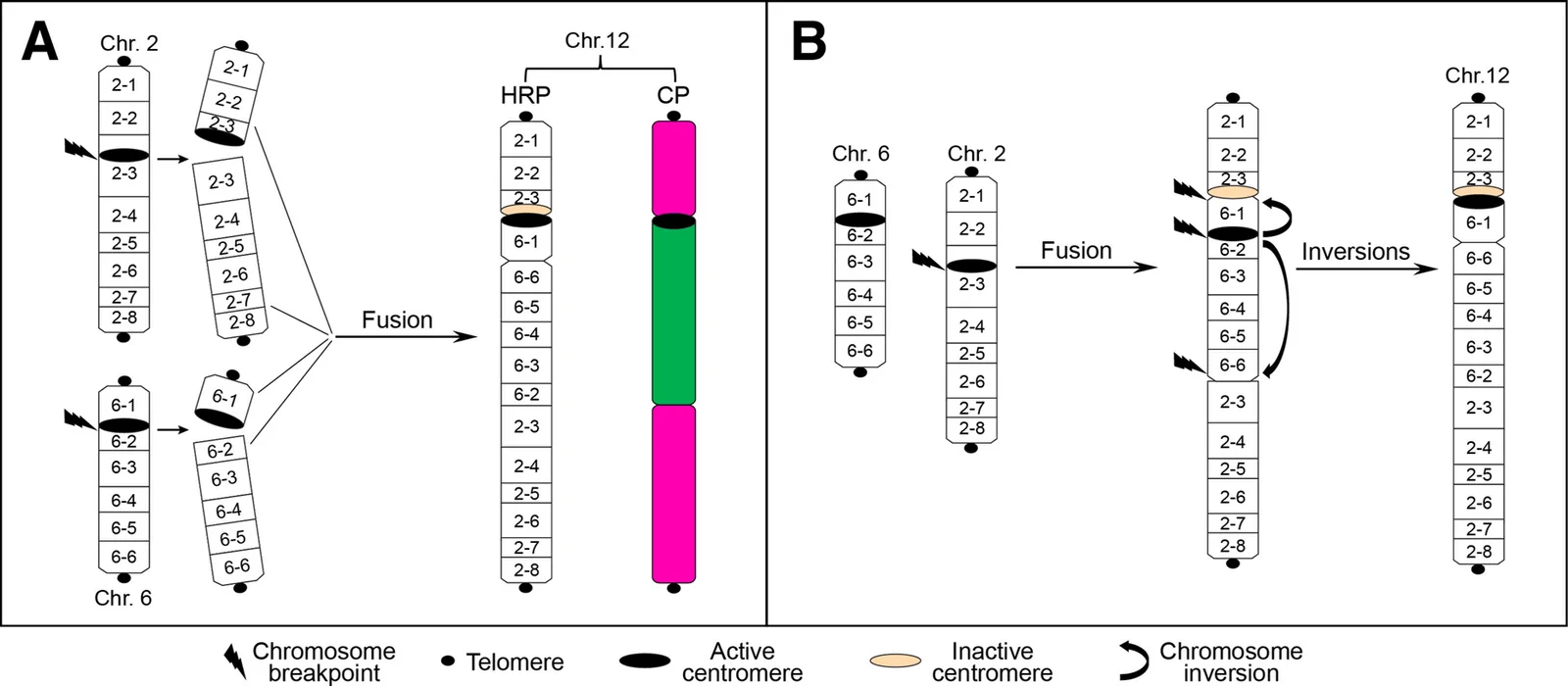
High-resolution regional painting (HRP) and possible mechanism for the origin of chromosome 12 in *Narenga porphyrocoma* and *Erianthus rockii.* Chromosome 12 originated from the fusion of ancestral chromosomes 2 and 6 (Yu et al. 2025). **A** A proposed mechanism based on centromere misdivision followed by fragment rejoining. Ancestral chromosomes 2 and 6 were subdivided into eight (2–1 to 2–8) and six (6–1 to 6–6) consecutive regions, respectively, using HRP probes. Centromere misdivisions of both chromosomes, followed by rejoining of the resulting chromosome segments, fully explain the observed order of the 12 regional painting signals on chromosome 12. The corresponding chromosome-painting (CP) pattern is shown at right. **B** Fusion mechanism based on the classical nested chromosome fusion (NCF) model. Although NCF can generate the same fusion chromosome, additional chromosome breaks and two subsequent chromosomal inversions are required to produce the observed order of the 12 HRP segments. Thus, HRP supports centromere misdivision followed by fragment rejoining as the most parsimonious mechanism for the origin of chromosome 12. Modified from Figure S7 of Yu et al. (2025)

First, HRP revealed that the fusion breakpoints were not randomly distributed but were consistently located in the proximal regions near the ancestral centromeres. All five fusion chromosomes in both species can be parsimoniously explained by a centromere misdivision followed by rejoining of the four resulting chromosome fragments (Yu et al. 2025). Although these fusion chromosomes could also be interpreted under the classical nested chromosome fusion (NCF) or end-to-end fusion (EEF) models (Lysak 2022), using the NCF and EEF models would require additional chromosome breakage events, resulting from double-strand breaks (DSBs) during meiosis, and subsequent chromosomal inversion(s) to account for the observed chromosome structures (Yu et al. 2025) (Fig. 2). Therefore, centromere misdivision followed by fragment rejoining provides the most parsimonious explanation for the origin of these fusion chromosomes.

Second, each fusion event was accompanied by the inactivation of one of the two ancestral centromeres. HRP analyses further revealed that centromere retention was not random. In every case, the functional centromere was derived from the ancestral centromere located closer to the center of the newly formed fusion chromosome (Fig. 2). Remarkably, no detectable remnants of the inactivated centromeres or the interstitial telomeric sequences generated by chromosome fusion were detected, suggesting their rapid elimination during subsequent chromosome evolution.

Third, *Miscanthus sinensis* (2n = 4x = 38), another allotetraploid species within the *Saccharum* complex, contains only a single fusion chromosome derived from ancestral chromosomes 4 and 7 (Mitros et al. 2020; Yu et al. 2022). Interestingly, an identical fusion chromosome is also present in both *N. porphyrocoma* and *E. rockii* (Yu et al. 2022). This observation suggests that the fusion chromosomes in *N. porphyrocoma* and *E. rockii* were likely generated through successive fusion events during evolution, rather than by a single evolutionary event that simultaneously produced all five fusion chromosomes within one subgenome.

The striking pattern of chromosome fusions affecting an entire subgenome in *N. porphyrocoma* and *E. rockii* raises several intriguing biological questions. To what extent have these extensive chromosome fusions reshaped genome organization and epigenome architecture? Have the fusion events contributed to the stabilization and allopolyploidization of these species? Does the subgenome containing the fusion chromosomes exhibit subgenome dominance, or does it behave similarly to the subgenome retaining the ancestral chromosome complement? Addressing these questions will provide important insights into the evolutionary consequences of large-scale chromosome restructuring in polyploid genomes.

#### Rare fixation of chromosomal translocations during evolution

Reciprocal chromosomal translocations represent one of the major classes of structural chromosomal rearrangements in eukaryotes. A heterozygous reciprocal translocation typically results in semisterility because adjacent segregation of the quadrivalent associated with translocated chromosomes during meiosis produces nearly 50% of unbalanced gametes, thereby imposing a substantial fitness cost on the carrier. Cytological stabilization requires the translocation to become homozygous. In animals, as well as in dioecious and self-incompatible plant species, this would require mating between two individuals carrying the same translocation, an event with an inherently low probability. The rarity of such matings, together with the severe fitness penalty associated with semisterility, explains why reciprocal translocations are seldom fixed in natural populations. Nevertheless, CCP studies, together with genome sequencing analyses, have uncovered several examples in which reciprocal translocations became fixed during plant evolution.

Potato and tomato diverged approximately 7.3 Mya (The Tomato Genome Consortium 2012). Strong chromosome synteny has been preserved across all 12 chromosome pairs of these two species (Braz et al. 2018; The Tomato Genome Consortium 2012). However, *Solanum etuberosum*, which diverged from the potato-tomato lineage approximately 8.3 Mya (Tang et al. 2022), carries a reciprocal translocation between chromosomes 2 and 7 (Braz et al. 2018). Similarly, two ancestral karyotypes have been recognized within the Brassicaceae supertribe Camelinodae, whose species have diverged for approximately 20 million years (Hendriks et al. 2023). These two karyotypes differ by a reciprocal translocation between ancestral chromosomes 7 and 8 (Jiang et al. 2025). In the Rutaceae, chromosome synteny is highly conserved among Aurantioideae species that diverged approximately 20 Mya, whereas three reciprocal translocations were identified in the more distantly related species *B. albiflora* (He et al. 2026b)*.*

These examples demonstrate that reciprocal chromosomal translocations are rarely detected among closely related species. However, comparative analyses show that reciprocal translocations can arise and become fixed over longer evolutionary timescales. A similar pattern is evident in mammals. Humans (2n = 46) and chimpanzees (*Pan troglodytes,* 2n = 48), which diverged approximately 7–8 Mya (Langergraber et al. 2012), differ primarily by the fusion that generated human chromosome 2 and show no evidence of reciprocal translocations. In contrast, the gorilla (*Gorilla gorilla*, 2n = 48), which diverged from the human lineage 8–10 Mya (Scally et al. 2012), possesses several reciprocal translocations relative to the human genome (Stankiewicz et al. 2001; Stanyon et al. 1992; Ventura et al. 2011).

The occurrence and fixation of reciprocal translocations in both plant and animal lineages raise a fundamental evolutionary question: how can a chromosomal rearrangement that initially imposes a substantial fitness cost spread through and eventually become fixed within a population? One intriguing possibility is that the translocation induces broader genomic or epigenomic changes that confer a selective advantage sufficient to outweigh the initial reproductive disadvantage. Alternatively, fixation may occur through demographic processes such as population bottlenecks or genetic drift in small populations. CCP studies across additional plant lineages will help identify suitable model systems for addressing these fundamental questions concerning chromosome evolution and speciation.

## Future perspectives

Over the past decade, oligo-based chromosome painting has evolved into a powerful platform for investigating chromosome biology in plants. Chromosome-specific painting in diverse plant lineages has enabled studies that were previously impossible, including chromosome-specific analyses of meiotic pairing, crossover formation, chromosome fusion, and karyotype evolution. As chromosome painting becomes established in additional plant lineages, new model systems will undoubtedly emerge for addressing fundamental questions concerning the structure, behavior, transmission, and evolution of individual chromosomes and entire genomes.

Chromosome identification and karyotype analysis will likely remain the most widespread applications of chromosome painting in the near future, particularly in plant groups that have not yet been explored cytogenetically. However, perhaps the most exciting future directions are the integration of chromosome painting with three-dimensional (3D) chromosome and genome biology. Oligo-based chromosome painting has already been used to visualize chromosome territories and the spatial organization of individual chromosomes in interphase nuclei (Albert et al. 2019; Dolezalová et al. 2024; Ning et al. 2024). Continued improvements in oligo-based 3D chromosome painting may enable quantitative analyses of chromosome architecture, including chromosome position, shape, volume, and degree of chromatin compaction.

At the same time, chromosome conformation capture technologies (3C and its derivatives, particularly Hi-C) have revolutionized our understanding of high-order genome organization by revealing chromatin interaction patterns, chromatin compartments, topologically associating domains (TADs), and chromosome territories. These sequencing-based approaches and oligo-based 3D chromosome painting provide fundamentally complementary information (Park et al. 2023; Patel et al. 2025). For example, comprehensive analyses using a combination of metaphase chromosome-based Hi-C data, oligo-FISH and super-resolution microscopy revealed that the sister chromatids of barley chromosomes are composed of chromatin helices of identical handedness. Each helical turn covers 20–40 Mb of DNA (Kubalová et al. 2023). Hi-C data can be used to infer chromatin structure from large populations of cells or chromosomes. By contrast, oligo-based 3D chromosome painting directly visualizes chromosome architecture in individual nuclei, making it possible to characterize chromosome organization at single-cell resolution and to reveal heterogeneity among cell types, tissues, developmental stages, and environmental conditions. Thus, oligo-based chromosome painting in plants will continue to evolve from a chromosome identification technique into an important tool for investigating chromosome and genome biology.

## Data Availability

No datasets were generated or analysed during the current study.

## Abbreviations

BAC: Bacterial artificial chromosome
CCP: Comparative chromosome painting
CO: Crossover
CP: Chromosome painting
EEF: End-to-end fusion
FISH: Fluorescence in situ hybridization
HRP: High-resolution regional painting
MI: Metaphase I
Mya: Million years ago
NCF: Nested chromosome fusion
Oligo: Oligonucleotide
PMC: Pollen mother cell
SNP: Single-nucleotide polymorphism
